# Efficacy and safety of office-based procedures for hemorrhoidal disease in patients with inflammatory bowel disease

**DOI:** 10.1007/s10151-025-03144-0

**Published:** 2025-05-08

**Authors:** T. Carvalho, T. Leal, B. Arroja, P. Mesquita, I. Malta, T. Gago, A. R. Gonçalves, R. Coelho, S. B. Ponte, P. Salgueiro, F. Castro-Poças, A. C. Bravo, C. Gouveia, A. M. Oliveira, M. Francisco, R. Gonçalves, A. C. Caetano

**Affiliations:** 1 Gastroenterology Department, Unidade Local de Saúde de Braga, Braga, Portugal; 2 Gastroenterology Department, Unidade Local de Saúde Gaia E Espinho, Gaia, Portugal; 3 Gastroenterology Department, Unidade Local de Saúde Algarve, Faro, Portugal; 4 Gastroenterology Department, Unidade Local de Saúde de São João, Porto, Portugal; 5 Gastroenterology Department, Unidade Local de Saúde de Santo António, Porto, Portugal; 6 Institute for the Biomedical Sciences Abel Salazar, Porto University, Porto, Portugal; 7 Gastroenterology Department, Unidade Local de Saúde de Loures-Odivelas, Loures, Portugal; 8 Gastroenterology Department, Unidade Local de Saúde de Amadora/Sintra, Amadora, Portugal

**Keywords:** Inflammatory bowel disease, Hemorrhoidal disease, Office-based procedures, Efficacy, Safety

## Abstract

**Background:**

Hemorrhoidal disease (HD) affects 2–20% of patients with inflammatory bowel disease (IBD) but treatment recommendations are scarce due to fear of higher morbidity in these patients. Currently, there is almost no data regarding nonsurgical treatment for HD in patients with IBD. This study aimed to evaluate the safety and efficacy of office-based procedures for HD in patients with IBD.

**Methods:**

A Portuguese multicenter retrospective study of patients with IBD undergoing office-based treatment for HD between July 2013 and December 2021 was performed. Data regarding the patients’ IBD (type, disease location, medication, endoscopic remission) and HD (clinical success, recurrence, complications) were analyzed.

**Results:**

A total of 129 patients were included, 90 with ulcerative colitis and 37 with Crohn’s disease. Only 55% of patients presented endoscopic remission and 18% were under biologics. Additionally, 77 patients underwent rubber band ligation (RBL), 44 underwent polidocanol injection, and 8 had both treatments, for a total of 304 procedures. The treatment of HD was effective in 88% of patients, of which 16% relapsed (mean follow-up time of 55.3 ± 34.5 months). Complications were described in 17 (5.6%) procedures, 2 (0.66%) requiring invasive treatment. There were no cases of suppuration, stenosis, or anal incontinence. Success, relapse, and complications were not associated with IBD features. Clinical success and recurrence were similar between RBL and polidocanol foam sclerotherapy (PFS). RBL had more complications than PFS (15.6% versus 2.6%).

**Conclusions:**

This study is the first to focus exclusively on office-based procedures for hemorrhoidal disease in patients with IBD, demonstrating similar efficacy to the general population and a low complication rate.

## Introduction

Hemorrhoidal disease (HD) is a common condition affecting up to 40% of the population [1, 2]. HD usually causes rectal bleeding and prolapse, but it can also cause anal discomfort or pain, mucus, and anal itching [2]. The Goligher classification (grade I–IV) grades HD on the basis of the degree prolapse [3]. Rubber band ligation (RBL) is the first-line office-based treatment for HD grades I–III and it is more effective than liquid sclerotherapy [4]. Recent evidence showed that the foam formulation of polidocanol provides good/excellent efficacy and safety, even when compared with RBL [5, 6].

Inflammatory bowel disease (IBD) is a chronic inflammatory disease affecting the gastrointestinal tract that includes two disorders: Crohn’s disease (CD) and ulcerative colitis (UC) [7]. IBD usually presents periods of activity and periods of remission, and the main symptoms are abdominal pain, diarrhea, and bloody stools [7, 8]. The latter can also be attributed to HD [9].

Regarding HD in patients with IBD, it seems less prevalent (2–20%) than the general population [8–11]. This can be partially explained by symptom overlap or the clinical focus mainly on IBD [8, 10], making HD underestimated and undervalued in these patients. The chronic diarrhea frequently presented by patients with IBD probably triggers HD, leading us to assume a greater prevalence than that currently described [12]. Treatment of HD in patients with IBD was traditionally cautious due to early reports of high complication rates and even some cases of proctectomy [13, 14]. Conversely, recent studies re-exploring perianal surgery in IBD described a low level of complications that led to a reassuring impact among the healthcare providers of patients with IBD [15, 16]. McKenna et al. found that surgical and nonsurgical treatment of HD and skin tags in IBD was safe, even in patients under biologics and without endoscopic remission [15].

Even though there are limited data regarding HD treatment in patients with IBD, there is even less information regarding nonsurgical treatment, since no studies are focusing solely on office-based procedures.

We aimed to assess the efficacy and safety of office-based treatment for HD in patients with IBD. Additionally, we intended to compare the efficacy and safety outcomes between the RBL and polidocanol foam sclerotherapy (PFS) groups.

## Methods

### Study design and patient selection

All adult patients with IBD who underwent office-based procedures for symptomatic HD at seven Portuguese centers between January 2013 and December 2021 were screened for eligibility. Patients were excluded if the HD diagnosis was before the IBD diagnosis or if they had active fistulizing perianal disease. Included patients were those who failed conservative treatment with lifestyle changes and phlebotonic medication before the office-based procedure, as recommended by current guidelines [1]. Treatments were performed on an outpatient basis and without any sedation. No prophylactic antibiotics were given. The type of office-based procedure was chosen by the assistant gastroenterologist, a specialist in proctology with experience in managing hemorrhoidal disease and performing office-based procedures, on the basis of availability, clinical expertise, and patient suitability.

### Data collection

Clinical records were reviewed retrospectively to obtain relevant factors, including age, gender, IBD characteristics, IBD medication concomitant with HD treatment (first session), IBD disease status, HD symptoms, Goligher classification, type of HD office-based procedure, number of sessions, clinical success, complications, time of follow-up, and surgery. Perianal disease associated with IBD was defined as fistulizing (e.g., perianal fistulas or abscesses) and non-fistulizing (e.g., fissures and ulcerations). Fistulizing perianal disease activity was defined as the presence of symptoms such as pain, discharge, active inflammation, or the need for medical or surgical intervention.

Endoscopic remission in CD was defined as a Simple Endoscopy Score for CD < 3 points or absence of ulcerations, and in UC as a Mayo endoscopic subscore of 0 points, according to STRIDE II consensus [17]. The endoscopic exam before the first office-based treatment was considered.

### Efficacy outcomes

The primary outcomes were clinical success and recurrence.

Clinical success was defined as a significant improvement of initial symptoms related to HD after the office-based procedures, without the need for further interventional treatment. Data regarding subsequent surgery for HD was collected and defined office-based treatment failure.

Recurrence was defined as a reappearance of significant symptoms related to HD without response to medical treatment and the need for new office-based procedures or surgery.

### Safety outcomes

The secondary outcome was the safety of office-based procedures.

Complications were divided between early (< 15 days) and late (> 15 days) complications and included bleeding and moderate-to-severe pain requiring medical observation, urinary retention, hemorrhoidal thrombosis, anal ulcer, perianal abscess, anal stricture and fecal incontinence (de novo). All complications were collected; therefore, more than one complication per procedure or per patient could be described during the treatment program.

### Sample size and statistical analysis

The sample size was calculated using the application G*Power® V3.1.9.7. It was based on IBD and HD prevalence and it was determined that a minimum of 100 patients should be included.

In the descriptive analysis, categorical variables were presented as numbers (*n*) and proportions (%). Continuous variables were described as means and standard deviations (SD) or medians and interquartile ranges (IQR) for skewed distributions. Quantitative variables were evaluated for normality using Kolmogorov–Smirnov test, combined skewness, and kurtosis measures.

The chi-squared test was used to compare categorical variables. Correlations were evaluated by calculation of either the Pearson or Spearman correlation coefficient, whether the variables exhibited a normal distribution, respectively.

To compare the two groups of patients, an independent *t*-test was used for normally distributed variables and the Mann–Whitney *U* test was used as a non-parametric alternative.

To compare office-based treatments, only patients undergoing exclusively RBL or PFS were considered, as those are the most common and effective treatments. Patients undergoing more than one type of treatment and liquid polidocanol was excluded from this analysis.

To evaluate independent predictors related to the event of interest, a binary logistic regression was applied in the multivariable analysis.

The statistical analysis was performed using IBM® SPSS® Statistics software, version 27. With a 95% confidence interval, a *p* < 0.05 was considered statistically significant.

### Ethics

The research protocol was approved by the Ethics Committee of ULS de Braga. Due to the retrospective nature of the study and the anonymity of clinical data, informed consent was waived.

## Results

### Patient cohort characteristics

A total of 129 patients were included; 90 patients with UC, 37 with CD, and 2 with unclassified IBD. Most patients with UC had proctitis (*n* = 42; 47.2%) and 20 patients (15.5%) with Crohn’s disease had colonic involvement. The two cases of perianal disease identified were specifically inactive fistulizing perianal disease, with no symptoms, such as pain, discharge, or active inflammation, for more than a year, and no requirement for medical or surgical intervention. Regarding medication for IBD, 23 patients (17.8%) were under biologics and 19 patients (14.7%) were under immunosuppressants. At the time of the first treatment, 71 patients (55%) had endoscopic remission.

Concerning HD, the most common symptom was rectal bleeding (*n* = 116; 89.9%), and most of patients had HD Goligher grade II (*n* = 62; 48.1%) or III (*n* = 42; 32.5%). During HD treatment, 77 patients (59.7%) underwent RBL, 44 patients polidocanol injection (34.1%), and 8 patients (6.2%) both treatments.

All patient characteristics are further summarized in Table 1.

**Table 1:** Baseline patient characteristics, as stratified by the type of office-based procedure (RBL or PFS)

	Total *N* = 129	RBL *n* = 77	PFS *n* = 39	*p*-value
Age (years), mean ± SD	49.3 ± 11.6	48.7 ± 11.6	55.0 ± 13.7	**0.010**
Sex, *n* (%)				
Male	77 (59.7%)	46 (59.7%)	23 (41%)	0.937
Female	52 (40.3%)	31 (40.3%)	16 (59%)	
IBD, *n* (%)				
Crohn’s disease	37 (28.7%)	25 (32.5%)	9 (23.1%)	0.256
Ulcerative colitis	90 (69.8%)	50 (64.9%)	30 (76.9%)	
Unclassified IBD	2 (1.6%)	2 (2.6%)	0 (0%)	
Montreal classification, *n* (%)				
**Crohn’s disease**				
A1: A2: A3	0 (0%):23 (62.2%):14 (37.8%)	0 (0%):17 (68%):8 (32%)	0 (0%):3 (33.3%):6 (66.6%)	0.070
L1: L2: L3	17 (45.9%):8 (21.6%):12 (32.4%)	10 (40%):5 (20%):10 (40%)	6 (66.7%):2 (22.2%):1 (11.1%)	0.256
B1: B2: B3	27 (73%):6 (16.2%):4 (10.8%)	20 (80%):2 (8%):3 (12%)	6 (66.7%):3 (33.3%):0 (0%)	-
**Ulcerative colitis**				
E1: E2: E3	42 (47.2%):30 (33.7%):17 (19.1%)	29 (59.2%):12 (24.5%):8 (16.3%)	11 (36.7.2%):13 (43.3%):6 (20.0%)	0.129
Inactive fistulizing perianal disease, *n* (%)	2 (1.6%)	2 (2.6%)	0 (0%)	–
IBD medication, *n* (%)				
No medication	15 (11.6%)	9 (11.7%)	6 (15.4%)	0.575
Mesalazine	86 (66.7%)	50 (64.9%)	27 (69.2%)	0.644
Budesonide	2 (1.6%)	2 (2.6%)	0 (0%)	-
Azathioprine/6-MP	19 (14.7%)	13 (16.9%)	4 (10.3%)	0.340
Biologic	23 (17.8%)	12 (15.6%)	8 (20.5%)	0.507
IBD duration (years), mean ± SD	10.0 ± 7.4	10.3 ± 7.9	7.1 ± 6.8	0.506
Endoscopic IBD remission, *n* (%)	71 (55%)	37 (48.1%)	27 (69.2%)	**0.016**
Anticoagulant or antiaggregant intake, *n* (%)	10 (7.8%)	3 (3.9%)	7 (17.9%)	**0.011**
Previous surgery for HD, *n* (%)	8 (6.2%)	4 (5.2%)	3 (6.8%)	0.704
HD symptoms, *n* (%)				
Rectal bleeding	116 (89.9%)	66 (85.7%)	37 (94.9%)	0.214
Prolapse	51 (39.5%)	28 (36.4%)	17 (43.6%)	0.451
Anal discomfort/pain	25 (19.4%)	12 (15.6%)	10 (25.6%)	0.192
Mucus discharge	18 (14%)	14 (18.2%)	2 (5.1%)	0.054
Anal itching	12 (9.3%)	10 (13%)	2 (5.1%)	0.189
Goligher classification, *n* (%) Grade I: II: III	25 (19.4%):62 (48.1%):42 (32.5%)	21 (27.3%):34 (44.2%):21 (28.5%)	4 (10.3%):18 (48.7%):16 (41%)	0.090
Officed-based procedures, *n* (%)				
Rubber band ligation	77 (59.7%)	–	–	–
Polidocanol	44 (34.1%)			
Liquid	5 (%)			
Foam	39 (%)			
Both	8 (6.2%)			
Number of procedures sessions (per patient), median (IQR)	2 (2)	2 (2)	1 (2)	0.080
Total number of officed-based procedures, *n*	304	180	75	–
Total number of rubber bands applied (per patient), median (IQR)	3 (2)	–	–	–
Clinical success, *n* (%)	113 (87.6%)	68 (88.3%)	33 (86.8%)	0.821
Recurrence, *n* (%)	18 (14.0%)	7 (10.3%)	7 (17.9%)	0.136
Complications (per patient), *n* (%)	16 (12.4%)	12 (15.6%)	1 (2.6%)	0.058
Early	14 (10.9%)	10 (13.0%)	1 (2.6%)	
Late	3 (2.3%)	2 (2.6%)	0 (0%)	
Complications (per procedure), *n* (%)	5.6%	6.6%	1.3%	–
Surgery for HD after office-based procedures, *n* (%)	14 (10.9%)	10 (13%)	4 (10.3%)	0.771
Time of follow-up after last session (months), mean ± SD	55.3 ± 34.5	60.1 ± 33.7	26.2 ± 18.1	** < 0.001**

*HD* hemorrhoidal disease, *IBD* inflammatory bowel disease, *IQR* interquartile range, *MP* mercaptopurine, *PFS* polidocanol foam sclerotherapy, *RBL* rubber band ligation, *SD* standard deviation

### Efficacy outcomes

Clinical success was achieved in 113 patients (87.6%). No specific IBD feature significantly affected clinical success, namely IBD type, disease location, IBD medication, and endoscopic remission (Table 2).

**Table 2:** Univariate analysis for clinical outcomes (success and recurrence)

	Univariate analyses					
	Clinical success			Recurrence		
	Yes *n* = 113	No *n* = 15	*p*-Value	Yes *n* = 18	No *n* = 95	*p*-Value
Age (years), mean ± SD	51.3 ± 12.6	49.6 ± 11.9	0.629	52.7 ± 13.5	51.0 ± 12.4	0.594
Sex (male), *n* (%)	68 (60.2%)	9 (60%)	0.990	12 (66.7%)	56 (58.9%)	0.540
IBD (ulcerative colitis), *n* (%)	79 (70.5%)	10 (71.4%)	> 0.999	15 (83.3%)	64 (68.1%)	0.194
Montreal classification, *n* (%)						
**Crohn’s disease**						
Colic involvement	18 (54.5%)	2 (50%)	> 0.999	1 (33.3%)	17 (56.7%)	0.579
**Ulcerative colitis**						
E1: E2: E3	36 (46.2%): 27 (34.6%): 15 (19.2%)	5 (50%): 3 (30%): 2 (20%)	0.958	6 (40%): 6 (40%): 3 (20%)	30 (47.6%): 21 (33.3%): 12 (19%)	0.855
Medication for IBD, *n* (%)						
Without medication	13 (11.5%)	1 (6.7%)	> 0.999	3 (16.7%)	10 (10.5%)	0.454
Mesalazine	75 (66.4%)	11 (73.3%)	0.772	13 (72.2%)	62 (65.3%)	0.567
Budesonide	2 (1.8%)	0	-	0	2 (2.1%	-
Azathioprine/6-MP	17 (15%)	2 (13.3%)	0.851	4 (22.2%)	13 (13.7%)	0.469
Biologic	21 (18.6%)	2 (13.3%)	0.609	2 (11.1%)	19 (20%)	0.518
IBD duration (years), mean ± SD	10.1 ± 7.5	10.0 ± 7.5	0.979	8.3 ± 6.8	10.4 ± 7.6	0.290
Endoscopic IBD remission, *n* (%)	62 (56.9%)	8 (53.3%)	0.789	10 (55.6%)	52 (57.1%)	0.901
HD symptoms, *n* (%)						
Rectal bleeding	102 (91.2%)	12 (80%)	0.180	17 (94.4%)	86 (90.5%)	> 0.999
Prolapse	41 (36.3%)	10 (66.7%)	**0.024**	7 (38.9%)	34 (35.8%)	0.802
Anal discomfort/pain	20 (17.7%)	5 (33.3%)	0.170	5 (27.8%)	15 (15.8%)	0.309
Mucus discharge	17 (15%)	1 (6.7%)	0.693	0 (0%)	17 (17.9%)	–
Anal itching	11 (9.7%)	1 (6.7%)	> 0.999	0 (0%)	11 (11.6%)	-
Number of symptoms, mean ± SD	1.7 ± 0.78	1.9 ± 1.0	0.306	1.6 ± 0.7	1.7 ± 0.8	0.566
Goligher classification, *n* (%)						
Grade I or II: III	80 (67.2%): 33 (29.2%)	6 (40%): 9 (60%)	**0.037**	13 (72.2%): 5 (27.8%)	67 (70.5%): 28 (29.5%)	0.885
Officed-based procedures, *n* (%) RBL: PFS	68 (67.3%): 33 (32.7%)	9 (64.3%): 5 (35.7%)	> 0.999	7 (50%): 7 (50%)	61 (70.1%): 26 (29.9%)	0.217
Complications (per patient), *n* (%)	14 (12.4%)	2 (13.3%)	> 0.999	3 (16.7%)	11 (11.6%)	0.695
Number of procedures sessions (per patient), median (IQR)	2 (2)	2 (3)	0.215	3 (4)	2 (2)	**0.079**
Total number of rubber bands applied (per patient), median (IQR)	3 (2)	3 (7)	0.349	4 (3)	2 (2)	0.436
Time of follow-up after last session, mean ± SD	47.3 ± 33.8	42.5 ± 34.8	0.607	59.1 ± 48.8	45.1 ± 30.0	0.254

*HD* hemorrhoidal disease, *IBD* inflammatory bowel disease, *IQR* interquartile range, *MP* mercaptopurine, *PFS* polidocanol foam sclerotherapy, *RBL* rubber band ligation, *SD* standard deviation

Clinical success was significantly lower in patients with prolapse symptoms (*p* = 0.024) and HD Goligher grade III (*p* = 0.037), compared with grades I or II. The type of treatment (RBL versus polidocanol) or the total number of procedures had no impact on clinical success (Table 2).

Looking at patients that reached clinical success, recurrence occurred in 18 patients (15.9%), during a mean follow-up of 55.3 ± 34.5 months. Similar to clinical success, no IBD-related characteristic affected the recurrence rate. (Table 2).

### Safety outcomes

A total of 16 patients (12.1%) had at least one complication during the treatment program. Of a total of 304 procedures, 17 were associated with complications (5.6%): 14 were early (4.6%) and 3 were late complications (1.0%). In nine procedures (3.0%), patients had significant pain after the procedure, six (2.0%) had hemorrhoidal thrombosis, two (0.66%) bled from the ligation scar, and one (0.33%) developed an anal ulcer. Only two complications (0.66%) needed invasive treatment, namely one thrombosed hemorrhoid that underwent surgery and one bleeding from a ligation scar that required endoscopic treatment. The remaining complications were treated conservatively. All complications had a favorable evolution.

Similar to the efficacy outcomes, no IBD-related characteristic was associated with complications, specifically IBD type, disease location, IBD medication, or endoscopic remission (Table 3).

**Table 3:** Univariate and multivariate analysis for safety outcomes (complications)

	Univariate analyses			Multivariate analyses		
	Complications					
	Yes *n* = 16	No *n* = 113	*p*-Value	OR	95% CI	*p*-Value
Age (years), mean ± SD	47.6 ± 10.2	51.7 ± 12.7	0.217	0.977	0.922–0.1035	0.430
Sex (male), *n* (%)	7 (43.8%)	70 (61.9%)	0.165	–	–	–
IBD (ulcerative colitis), *n* (%)	13 (81.3%)	77 (69.4%)	0.394	–	–	–
Montreal classification, *n* (%)						
**Crohn’s disease**						
Colic involvement	2 (66.7%)	18 (52.9%)	> 0.999	–	–	–
**Ulcerative colitis**						
E1: E2: E3	6 (50%):3 (25%):3(25%)	36 (46.8%):27(35.1%):14 (18.2%)	0.745			
Medication for IBD, *n* (%)						
Without medication	3 (18.8%)	12 (10.6%)	0.399	–	–	–
Mesalazine	11 (68.8%)	75 (66.4%)	0.850			
Budesonide	0	2 (1.8%)	-			
Azathioprine/6-MP	2 (12.5%)	17 (15%)	> 0.999			
Biologic	1 (6.3%)	22 (19.5%)	0.302			
IBD duration (years), mean ± SD	8.25 ± 6.84	10.3 ± 7.51	0.310	–	–	–
Endoscopic IBD remission, *n* (%)	8 (53.3%)	63 (57.3%)	0.773	1.859	0.500–6.909	0.355
HD symptoms, *n* (%)						
Rectal bleeding	13 (81.3%)	103 (91.2%)	0.205	–	–	–
Prolapse	9 (56.3%)	42 (37.2%)	0.144			
Anal discomfort/pain	7 (43.8%)	18 (15.9%)	**0.015**			
Mucus discharge	2 (12.5%)	16 (14.2%)	> 0.999			
Anal itching	2 (12.5%)	10 (8.8%)	0.644			
Number of symptoms, mean ± SD	2.06 ± 0.93	1.68 ± 0.77	0.074	–	–	–
Goligher classification, *n* (%)						
Grade I or II: III	7 (43.8%):9(56.3%)	33 (29.2%):80 (70.8%)	**0.031**	3.297	0.919–11.833	0.067
Officed-based procedures, *n* (%)						
RBL: PFS	12 (92.3%):1(7.7%)	65 (63.1%):38 (36.9%)	**0.058**	**10.464**	**1.137–96.346**	**0.038**
Number of procedures sessions (per patient), median (IQR)	2 (3)	2 (2)	0.277	–	–	–
Total number of rubber bands applied (per patient), median (IQR)	3 (2)	3 (2)	0.462	–	–	–
Time of follow-up after last session, mean ± SD	41.1 ± 32.2	47.2 ± 34.3	0.502	0.989	0.968–1.010	0.296

*CI* confidence interval, *HD* hemorrhoidal disease, *IBD* inflammatory bowel disease, *IQR* interquartile range, *MP* mercaptopurine, *OR* odds ratio, *PFS* polidocanol foam sclerotherapy, *RBL* rubber band ligation, *SD* standard deviation

There was a higher rate of complications in patients with HD Goligher grade III (*p* = 0.031), and patients who underwent RBL versus polidocanol (liquid and foam) (*p* = 0.030) (Table 3).

### Rubber band ligation versus polidocanol foam

A total of 77 patients underwent only RBL and 39 patients PFS. The RBL group was significantly younger (mean age 48.7 ± 11.6 years versus 55.0 ± 13.7 years; *p* = 0.010), with fewer anticoagulants/antiaggregant intake (3.9% versus 17.9%; *p* = 0.011), less endoscopic remission (55.3% versus 79.5%; *p* = 0.006), and a longer follow-up (mean time 60.1 ± 33.7 months versus 26.2 ± 18.1 months; *p* < 0.001) (Table 1).

The clinical success and recurrence were similar between the RBL and PFS groups (88.3% versus 86.6%; *p* = 0.821; 10.3% versus 17.9%; *p* = 0.136, respectively). There was a trend toward higher complications in the RBL group (*p* = 0.058).

In a multivariate analysis, the type of treatment (RBL versus PFS) was an independent predictor of complications (OR 10.464, 95% CI 1.137–96.346, *p* = 0.038), adjusted for confounding variables such as age, endoscopic remission, and time of follow-up (Table 3).

## Discussion

This is the largest study evaluating office-based procedures for HD in patients with IBD to date and it reports the same efficacy and safety of office-based treatments as the general population.

In patients without IBD, various studies describe high rates of efficacy (83–98%) for both RBL and injection sclerotherapy, mainly PFS [4–6, 18–20]. In our cohort of patients IBD, we found similar results, with an overall success rate of 87.6%.

The recurrence rate described in studies in the general population varies widely in terms of time definitions and type of treatment, but there are reports of 10–17% at 3-month to 5-year follow-up [4, 18]. There are conflicting results when comparing RBL and sclerotherapy since sclerosant agents have different efficacy outcomes. Salgueiro et al. reported a lower recurrence rate with PFS than RBL (16.1% versus 41.2%) [5]. In our study, we observed 16% of recurrence in a mean follow-up time of 55 months, which is comparable to the results previously described in patients without IBD.

Studies addressing HD treatment in patients with IBD focus mostly on safety outcomes, not evaluating the impact of IBD characteristics on efficacy outcomes. In our study, there was no association between IBD features, such as IBD type, disease location, medication or endoscopic remission, and clinical success or recurrence. Remarkably, only HD characteristics were associated with lower clinical success, namely symptoms of prolapse and Goligher grade III; predictors of treatment failure also observed in the general population [19].

Nowadays, there are no guidelines regarding a stepwise treatment for HD in patients with IBD due to lack of data in this population [1, 21, 22]. The first studies published in the 1970s and 1980s regarding hemorrhoidectomy and perianal surgery in patients with IBD described a high risk of complications, including proctectomy. Those authors associated surgical complications with impaired wound healing, mainly in patients with CD [13, 14]. A review including 99 patients with CD who underwent hemorrhoidectomy reported high morbidity, with 17% complications, including fistulas, sepsis, and fecal incontinence [11]. However, this review included older studies, and not all studies can properly associate all complications to HD treatment since some patients had severe active disease. In patients with UC, only 5.5% of complications were observed, including one anorectal stenosis and one proctectomy. In addition, this study concluded that complications were more frequent in patients with unknown IBD, probably due to uncontrolled disease and more aggressive surgery [11]. All the reports of complications, such as perianal sepsis, fecal incontinence, anal stenosis, fistulas, and the need for proctectomy, worried the medical community since these complications were extremely rare in the general population. Since then, most physicians avoided invasive procedures in patients with IBD.

Nevertheless, there are now some studies indicating that surgery is relatively safe in these patients [15, 16, 23–25], especially when the disease is in remission and without active perianal disease.

Regarding office-based treatments, scarce data were compiled in a meta-analysis of HD surgical and nonsurgical treatment in IBD, including 222 patients. Of all published studies, a total of 39 patients underwent RBL, and one anal stenosis was reported as a complication. Considering all types of treatments, there was a complication rate of 9%, however, patients with CD had twice as many complications than patients with UC. The complications reported were anal abscess, fistula, urinary retention, anal stenosis, nonhealing wound, and anal fissures [26].

In our cohort, there were no reports of suppuration, stenosis, or anal incontinence. There was a 5.6% complication rate, none severe, and all were usually described in patients without IBD. It should also be emphasized that only 0.66% of complications needed invasive treatment and all complications had a favorable evolution. Additionally, the authors highlight that no patient underwent office-based treatment with active perianal disease.

A prospective study including 120 patients without IBD who underwent office-based procedures reported 20% of complications, however, 18% were minor complications. Only 2% had moderate complications (hemorrhoidal thrombosis requiring drainage and bleeding, not requiring blood transfusion or surgery), without cases of severe complications [5]. Considering moderate-to-severe complications, our results were similar to those of the general population.

In most IBD studies there is no reference to endoscopic remission or medication at the time of HD treatment. Mckenna et al. reported an endoscopic remission rate of 45% and biologic treatment in 20% of patients, without complications associated with RBL, performed in 35 patients [15]. In our cohort, 18% were under biologics and 15% were under immunosuppressants, with an endoscopic remission of 55%. There was no association between IBD-related features and a higher rate of complications. However, it is important to emphasize that patients with active perianal disease were not included. Recent studies hypothesized that modern-era IBD treatments are associated with improved disease control and mucosal healing and can partially explain the better results shown in the last years [15, 16].

Our data suggest that the clinical success and recurrence were similar between RBL and PFS. However, the type of treatment (RBL versus PFS) was an independent predictor of complications, higher in the RBL group. The authors highlight that there were no severe complications in both groups. Looking at other studies comparing both techniques, Salgueiro et al. found that PFS was more effective than RBL in reaching complete therapeutic success, with a lower rate of recurrence and complications [5]. Kanellos et al. also supported that sclerotherapy has significantly fewer complications than RBL [27].

The main limitation of our study is its retrospective design, which can underestimate complications, particularly mild ones. Nevertheless, it was possible to include all complications that needed a medical evaluation. Due to the study design, it was not possible to apply validated scales to measure clinical success and recurrence more accurately. Another limitation is the potential difficulty in distinguishing rectal bleeding caused by hemorrhoidal disease from that due to active ulcerative colitis or Crohn’s disease. To address this, we carefully evaluated clinical history, associated symptoms, and anoscopic findings, and prioritized the use of rectosigmoidoscopy when uncertainty remained.

The strengths of this study include the relatively large cohort size compared with existing studies and the extended follow-up period, which provide meaningful insights into the outcomes of office-based procedures in this specific patient population.

While this study is, to our knowledge, the first to exclusively evaluate office-based treatments for hemorrhoidal disease in patients with IBD and to compare RBL and PFS in this subgroup, we acknowledge the need for further research in this area. This includes prospective studies with standardized classifications and terminology to allow for better comparison between patient groups, as well as studies addressing hemorrhoidal management in patients with active fistulizing perianal disease. Additionally, a better understanding of patient perspectives on management options and outcomes could provide critical insights to improve evidence-based recommendations for this unique population. 

Our findings, demonstrating similar efficacy to the general population with a low complication rate, suggest that office-based treatments are a safe and effective option for hemorrhoidal disease in patients with IBD and should be considered in clinical practice.

## Data Availability

The data that support the findings of this study are available on request from the corresponding author, Ana Célia Caetano. The data are not publicly available since it can contain information that could compromise the privacy of research participants.
